# Influence of Disease-Causing Mutations on Protein Structural Networks

**DOI:** 10.3389/fmolb.2020.620554

**Published:** 2021-03-10

**Authors:** Vasam Manjveekar Prabantu, Nagarajan Naveenkumar, Narayanaswamy Srinivasan

**Affiliations:** ^1^ Molecular Biophysics Unit, Indian Institute of Science, Bangalore, India; ^2^ National Centre for Biological Sciences, TIFR, Bangalore, India; ^3^ Bharathidasan University, Tiruchirappalli, India

**Keywords:** disease-causing mutations, protein structure networks, allostery, network variability, protein function

## Abstract

The interactions between residues in a protein tertiary structure can be studied effectively using the approach of protein structure network (PSN). A PSN is a node-edge representation of the structure with nodes representing residues and interactions between residues represented by edges. In this study, we have employed weighted PSNs to understand the influence of disease-causing mutations on proteins of known 3D structures. We have used manually curated information on disease mutations from UniProtKB/Swiss-Prot and their corresponding protein structures of wildtype and disease variant from the protein data bank. The PSNs of the wildtype and disease-causing mutant are compared to analyse variation of global and local dissimilarity in the overall network and at specific sites. We study how a mutation at a given site can affect the structural network at a distant site which may be involved in the function of the protein. We have discussed specific examples of the disease cases where the protein structure undergoes limited structural divergence in their backbone but have large dissimilarity in their all atom networks and vice versa, wherein large conformational alterations are observed while retaining overall network. We analyse the effect of variation of network parameters that characterize alteration of function or stability.

## Introduction

The amino acid sequence determines the protein 3-D structure (Anfinsen, 1973) which is related to its function. An alteration in the amino acid sequence can bring about changes in the folding and stability of the protein (Lorch et al., 1999; Lorch et al., 2000), interaction of the protein with other molecules (Rignall et al., 2002; Ung et al., 2006) and change in functional levels (Tiede et al., 2006) or overall function of the protein as well. A mutation in the amino acid sequence may alter the structure of a protein but it does not necessarily alter its function, although, the mutation at specific sites such as conserved residues can bring about a change in the structure and function of the protein.

In humans, the most frequent genetic variants are single nucleotide polymorphisms (SNPs) which have been studied extensively (Buetow et al., 1999; Cargill et al., 1999; Collins et al., 1999; Halushka et al., 1999). SNPs could be non-synonymous which bring about a change in the amino acid sequence. Several such genetic variants are known to cause mutations in their gene product and their information is available in resources such as the SNPdb (Sherry et al., 2001) and 1000 Genomes project (Auton et al., 2015). Some of the mutations in a protein are known to enhance the susceptibility or predisposition to a disease and are referred to as disease causing mutations. A few resources are available that map the gene variants to the diseases they may cause. ClinVar is a public archive mapping sequence variants and human phenotype (Landrum et al., 2018), COSMIC is a large catalogue of mutations associated with cancer (Forbes et al., 2017) and SwissVar is a one stop database for the easy retrieval of amino acid polymorphisms and the phenotype information (Mottaz et al., 2010). All the information from the SwissVar is now directly available via the UniProt knowledgebase (Bateman, 2019). However, specific information of the gene variants is compiled as a catalogue and is available on the Humsavar knowledge base which is an index of manually curated human polymorphisms and disease mutations. (https://www.uniprot.org/docs/humsavar).

Mutations in the protein sequence can alter the structure that is natively conferred by the sequence of the wildtype (Taverna and Goldstein, 2002; Tokuriki and Tawfik, 2009). In several scenarios the site of mutation is distant from the site of function, and still one observes a loss of function or alteration in functional levels (Mitternacht and Berezovsky, 2011; Yang et al., 2016). Although, the conformation of the mutant protein may be highly similar to the conformation of the wildtype, there could be alterations in their topologies at sites distant from the site of mutation (Rajasekaran et al., 2017). This concept of alteration of the structure at distant sites from the site of perturbation has been well documented under the subject of allostery (Gunasekaran et al., 2004; Weinkam et al., 2013; Naganathan 2019). Without much change in the overall topology of the protein an allosteric signal can transmit the effect of a perturbation to a different site in the protein structure (Guarnera and Berezovsky, 2019a; Guarnera and Berezovsky, 2019b). The internal protein structural network defines the connectivity between atoms/residues (Vijayabaskar and Vishveshwara, 2010). When perturbations are bought into the system such as disease-causing mutations, it is seen that the variation in the connectivity of the elements within the system brings about allosteric changes in functional sites and elsewhere (Dubay et al., 2015; Guarnera et al., 2017; Tan et al., 2019; Tee, Guarnera and Berezovsky, 2019; Guarnera and Berezovsky, 2020).

In this study, we use the Humsavar knowledge base to identify disease-causing mutations in proteins and analyse the variability in protein structural networks between wildtype and disease-causing variant. We explore the possibility of mutations at a given site that can affect the structural network at a distant site which may be involved in the function of the protein.

## Materials and Methods

### A Dataset of Disease-Causing Variants in Humans

The disease variant information provided in the Humsavar knowledge base is a manually curated subset of UniProtKB/Swiss-Prot protein data for human polymorphisms and disease mutations with their amino acid variations imported from Ensembl variation databases. Humsavar knowledge base has been screened to identify proteins that have X-ray crystal structures of the wildtype and associated disease-causing mutant available on the protein databank (PDB) (Berman et al., 2000; Berman et al., 2002). Of the 2,943 proteins reported on the knowledge base having disease causing variants, 1,316 of them have at least one crystal structure available. In the protein structural networks involved in our analysis we are looking into the geometry at local sites which are closer than 4.5 Å while constructing all atom networks (Yao et al., 2019). Hence, in our data set for analysis we have applied a resolution cut-off criterion of 3Å. Additional condition of a difference in refinement factors (R_free_−R_work_) of no more than 5% was also used. Protein structures available in the free form, without a bound ligand are chosen by screening them using the BioLip database (Yang et al., 2013). Disease cases are identified by pairwise alignment of the sequences obtained from uniport and PDB entries to obtain unique chains of disease-causing mutant and wildtype structures having the best resolution. 74 cases with crystal structures of the wildtype and corresponding disease-causing mutant are found. Details of these protein structure pairs are listed in Supplementary Table S1.

### All Atom - Protein Structure Network Model

The Protein Structural Network (PSN) models residues as nodes and constructs edges between nodes that satisfy the proximity criteria. Atoms from a pair of non-adjacent residues that fall within a distance cut-off of 4.5 Å are considered to make atom contact and therefore form an edge between the corresponding residues in the PSN (Brinda and Vishveshwara, 2005). The network model is an all-atom based, weighted and non-directed graph where the edge weight is given by:

$$Edgeweight\left(I_{ij}\right)=\frac{number\;of\;atom\;contacts\;between\;the\;residues\;i,\;j}{Highest\;number\;of\;atom\;contacts\;between\;the\;amino\;acids\;i,j}$$

The highest number of atom contacts between any pair amino acids is generated from analysing all the structures in the dataset of high-resolution crystal structures. In this paper, the Cα-atom position is used to represent the position of a node corresponding to a residue and edges are represented using lines. A hub is a node in the network that is well connected to several other nodes (Cohen and Barabási, 2002). We identify the minimum number of edges necessary to define at least one hub in all the structures of the disease cases and hence defined any node in the PSN having equal to or greater than 11 edges as a hub. We represent the hubs using spheres.

### Network Dissimilarity Score

The network dissimilarity score (NDS) iis used to compare two networks with identical number of nodes to generate a difference score that quantifies the dissimilarity in their spectra and the weight of edges (Gadiyaram et al., 2017; Ghosh et al., 2017). The adjacency matrix is a representation of a network which is generated as described in the *All Atom - Protein Structure Network Model*. Let us say we are comparing the networks of a proteins A and B. The adjacency matrices of PSN A and PSN B are compared to generate the edge difference score (EDS).

$$EDS=\frac{||A-B|{|}_{F}}{\sqrt{\left({\sum edge\;weight_{A}}^{\text{​ }}\times \sum edge\;weight_{B}\right)}}$$

The edge difference score captures the difference in edge weights between corresponding edges of the networks. A Laplacian of the adjacency matrix is derived before their spectra (eigen values and eigen vectors) are generated. The spectral information is used in computing the correspondence score (CRS) and eigen value weighted cosine scores (EWCS).

$$CRS=1-\frac{6{\sum {\left(Index\;Evec_{A}-Index\;Evec_{B}\right)}^{2}}^{\text{​ }}}{n\left(n^{2}-1\right)}$$

Where, n is number of nodes in the PSN. The index difference of eigen vectors, once arranged in ascending order of their eigen values, is used in the numerator.

$$EWCS=\frac{{\sum {(1-cosine\left(\theta_{ij}\right))}^{2}\left|1-Eval_{A}\right|\left|1-Eval_{B}\right|}^{\text{​ }}}{{\sum \left|1-Eval_{A}\right|\left|1-Eval_{B}\right|}^{\text{​ }}}$$

where, $Eval_{A}$ and $Eval_{B}$ are eigen values of PSN A and PSN B. The cosine between a pair of nodes is generated using the ratio between the dot product of their eigenvectors and the product of their magnitudes. The spectral comparison scores capture the local and global clustering of the nodes in the network. The components are formulated in computing the NDS:

$$NDS=\sqrt{EDS^{2}+EWCS^{2}+{\left(1-CRS\right)}^{2}}$$

An in-house python program is used to calculate the NDS in any pair of networks.

The NDS between the PSNs of the wildtype and mutant chain is generated.

NDS ranges from 0 (indicating absolute congruency/identical networks) to a score of √3 (indicating absolute dissimilarity to the extent of no match between the networks). TM-align tool is employed to generate structure based sequence alignment and structural difference information (Zhang and Skolnick 2005).

### Evaluating the Effect of Allostery

In order to study the effect of a perturbation such a disease-causing mutation on the structure of protein, the AlloSigMA server is employed. The server implements a structure-based statistical mechanical model of allostery, abbreviated SBSMMA (Guarnera and Berezovsky, 2016), to quantify the allosteric response that is communicated due to the effect of a perturbation like a molecular binding event or a mutation. The wildtype crystal structure of the protein being analysed is submitted as input to the server and an UP mutation perturbation is introduced. In this case, An UP mutation simulates the effect of mutation to a bulkier residue at the site of the disease-causing mutation. Crystal structures that had missing residues were completed using SWISS-MODEL (Guex and Peitsch, 1997). The AlloSigMA server results in an output of the response free energy of each residue that is accountable for the allosteric signal initiated by the mutation.

## Results

The perturbation in the structure of a protein due to disease causing mutations can be studied extensively using their native structural topologies (Ambrus et al., 2015; Ambrus et al., 2016; Szabo et al., 2018). It is understood that the resulting structural change manoeuvres the function or functional levels of the protein that is related to the onset of a disease. Here we study such variations in terms of structural networks of wildtype and disease related mutant. For the analysis, we identified proteins with disease-causing mutational variants from the Humsavar database and their corresponding wildtype and mutant crystal structures from PDB. We identified crystal structure variants corresponding to 74 disease cases and used those structures solved with the best resolution. The effect of mutations on their structure and network is analysed.

### Analysis of Protein Structural Network

Protein structure networks are a node-edge representation of the protein structure that efficiently displays the connectivity between different elements of their tertiary structure. Several studies in the past have made use of protein structure networks in studying the connectivity between residues based on features such as their spatial proximity and energy of interaction. We have used an all-atom network model to generate structural network information at the residue level with edges made between residues that are spatially proximal. Two residues are linked with an edge if a pair of their atoms is situated within a distance of 4.5 Å. The strength of the edge depends on the number of such atom pairs between the residues that are forming an edge. We have discussed the criteria for defining an edge in the *Methods* section. We generated the all-atom protein structural networks for all the individual chains of the wildtype and mutant protein structures in our dataset.

The alteration of the connectivity that arises as a result of mutation is studied by comparing the PSNs of the wildtype and the corresponding mutant. The variation in their connectivity is observed by segregating the edges into those that are retained and those that are unique to wildtype or mutant structures (Supplementary Figure S1). This means that the edges found to be unique to the wildtype structure are lost in the mutant. Similarly, those edges that are unique to the mutant structure are considered to be gained. The information of edges lost and gained in the wildtype PSN and mutant PSN is presented in Supplementary Figure S2A. Every wildtype and mutant structure in the dataset have at least one edge that is unique to it. Of the disease cases that are studied in the dataset, in 28 cases the wildtype has more unique edges than the mutant and in 45 disease cases the mutant has more unique edges. This suggests that in a majority of the disease cases more edges are gained than those that are lost. Only in the case of the cAMP-dependent protein kinase α catalytic subunit that is responsible for primary pigmented nodular adrenocortical disease (by mutation L206R) it is found that the number of edges lost in the wildtype is equal to the number of edges that are gained in the mutant. The wildtype and mutant in this disease case have 1,264 edges, 1,218 of these are retained while the remaining are lost and gained.

The information stored in the protein structure networks are predominantly in their edges and their connectivity. In order to study how well each element of the PSN is connected, we employed the use of a few basic network parameters such as the degree and strength of the nodes in the network. The number of edges that connect to a node constitutes its degree and the sum of all the edge weights connecting to a node spans the strength of each node. It is possible for a node to not form an edge with any other node; such a node is isolated in the network. Alternatively, a node can be well connected with other nodes of the network and form hubs. Hubs are elements in the network that are generally crucial since they are well connected to many other nodes. Perturbations in these nodes can have a more significant effect on the network than those nodes that are not hubs. Nodes from the PSNs in the dataset are found to have a maximum degree ranging from 11 to 18 as shown in the Supplementary Figure S3, hence for this analysis we have chosen to consider any node with a degree 11 or higher as a hub node, this ensures that each structure in our dataset is composed of at least one hub.

We observe variability in the number of hubs between the wildtype and mutant crystal structures (Supplementary Figure S4). Hubs that are retained in between the conformers are an indication of preserved local networks and retained structure around them. Hubs that are unique to the wildtype and mutant are also identified. Those hubs that are specific to the wildtype structure are lost in the mutant structure and the hubs unique to the mutant are gained. In 37 disease cases the number of hubs lost in the wildtype is greater than the number of hubs that are gained in the mutant and in 28 disease cases the number of hubs gained in the mutant are greater than those lost in the wildtype. In nine other disease cases the number of hubs unique to the wildtype and mutant are equal. There is no loss or gain of hubs in three disease cases. The highest number of hubs lost in wildtype structures is 32 and the highest number of hubs gained in the mutants is 23. The number of hubs unique to the wildtype structure and the number of hubs unique to the mutant are shown as a scatter in the Supplementary Figure S2B. The distribution of the number of hubs in the structures of our dataset can be found in the Supplementary Figure S4, S5. The functional relevance of the change in number of hubs has been discussed in detail for specific cases in a later section.

### Local Site Variation of Structural and Network Parameters

Change in degree of a residue between wildtype and the mutant suggests loss or gain of edges. The strength of an edge (edge weight) that connects two nodes may also change in the mutant. It is expected that a node corresponding to a residue which is buried in the protein structure has high degree and strength since they are in the proximity of several other nodes of the network. We have analysed the variation of network and structure parameters across the topologically equivalent residues and nodes. Since the focus of this work is on the mutation site that brings about the perturbation in the network and structure of the protein that may affect the functional sites, we have focused on studying the variability at these local sites in detail.

The change in degree and strength at the site of mutation reflects the change in local network at the site of perturbation. The change in sidechain atoms of the residue at the site of mutation plays a significant role in its degree that may or may not change in the PSN. For example, the highest gain in degree is in the case of apoptosis inducing factor where a glycine is mutated to a glutamate residue and the degree increases by 5. Likewise, when a phenylalanine is mutated to a serine in the case of Lysine-specific histone demethylase the degree at the site of mutation reduces by 7. The information of the change in degree and solvent accessibility at the site of mutation is shown in Supplementary Figure S5. In the dataset we find that at 11 mutation sites the mutated residue undergoes change in solvent accessibility. It is more common to see the mutation site buried in the wildtype whereas in the mutant state they are exposed since at 9 of the 11 sites we observe a buried residue get exposed in the mutant.

Using the information of active site and binding sites available in the Uniport database we identified 151 functional sites in the dataset and analysed the change in network parameters at these sites. The information of the change in degree at the functional site is shown in Supplementary Figure S6. No change in degree is observed at majority of the functional sites. The variation of degree at the functional site (ranges from loss of four edges to gain of four edges) is lower as compared to the variation of degree at the mutation sites (ranges from loss of seven edges to gain of five edges). In the dataset, only in the case of Septin-12 protein it is found that a mutation occurs at a site of function, where a threonine that is known to bind to GTP (Castro et al., 2020) is mutated to methionine (T89M) and the degree at the site changes from six in the wildtype to two in the mutant.

### Global Structural and Network Variation in the Crystal Conformers

**FIGURE 1:**
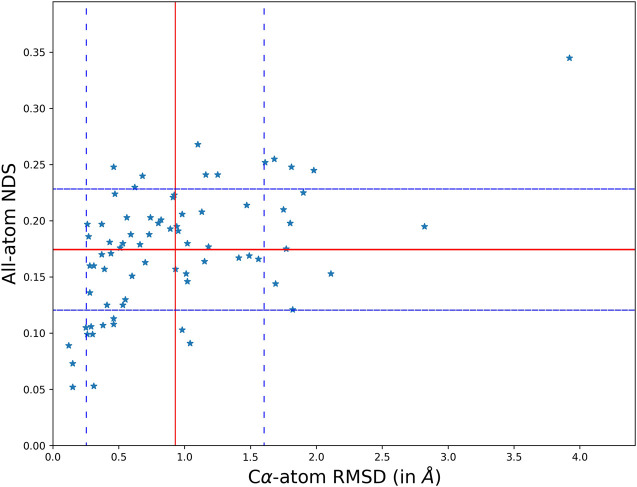
A scatter plot comparing the structural topology (Cα positions) and PSN of the wildtype and mutant using RMSD and NDS respectively. The comparison scores for each disease case are plot on the scatter. It is found that the structural divergence and network dissimilarity do not share strong linear relationship.

The overall variability in the crystal structures when the protein undergoes a disease-causing mutation has been studied by comparing their structures and networks separately. The structural difference between the conformers is calculated using the root mean square deviation (RMSD) that measures the divergence in the backbone topologies. In order to quantify the variation in the protein structure networks (PSN), a spectral comparison tool that is referred to as the NDS (network dissimilarity score) is used. The spectral comparison method quantifies the extent of dissimilarity between two networks with identical number of nodes. Only those residues that are topologically equivalent are identified by structural alignment and used for the comparison. All the structural and network comparison scores between the wildtype and mutant crystal structures in the dataset is generated using information of their coordinates. Figure 1 shows the scatter plot between Cα-atom RMSD and all-atom NDS.

The scatter of comparison scores suggests that the variation in the network is not strongly correlated to the variation of their structural topologies. The mean and standard deviation in the scores is plot on the scatter using red and blue (dotted) lines respectively. The mean NDS of the disease cases is 0.175 and the mean RMSD is 0.92 Å. A dataset of all pairs of available wildtype structures is used as a control in analysing the significance of the observed variability. In the control dataset the mean NDS is 0.12 and the mean RMSD is 0.57 Å which is relatively lesser than the variability in the disease cases (Supplementary Figure S7). It should be noted that RMSD and NDS plotted correspond to Cα positions and all atoms (including sidechains) respectively. Near absence of correlation in Figure 1 also conveys the message that there are examples with Cα positions well retained between wildtype and the mutant while the sidechain orientations are altered. There are also cases where the sidechain connectivity in networks are highly similar between wildtype and the mutant, but Cα trajectory has undergone a significant change.

### Specific Cases of Network and Structure Variability

In the global analysis of protein structure and network variability, we find several cases where the structural topology (Cα positions) is preserved but the all-atom network have changed considerably and the vice versa. In the first type of cases, the network variability is high, NDS is greater than the mean and standard deviation, even though the structures are well superimposed with lower than mean RMSD. In the second type of cases, the networks are not strongly dissimilar i.e. NDS lower than the mean of the dataset, but the structural difference suggests that they might not be as well preserved as their networks with RMSD greater than the mean and standard deviation of the dataset. Three disease cases from the dataset that fall into each of these categories are studied in detail.

### Network Variable Cases

#### Disease Mutation in Medium-Chain Specific Acyl-CoA Dehydrogenase (MCAD) Alters Local Network at the Functional Site

**FIGURE 2:**
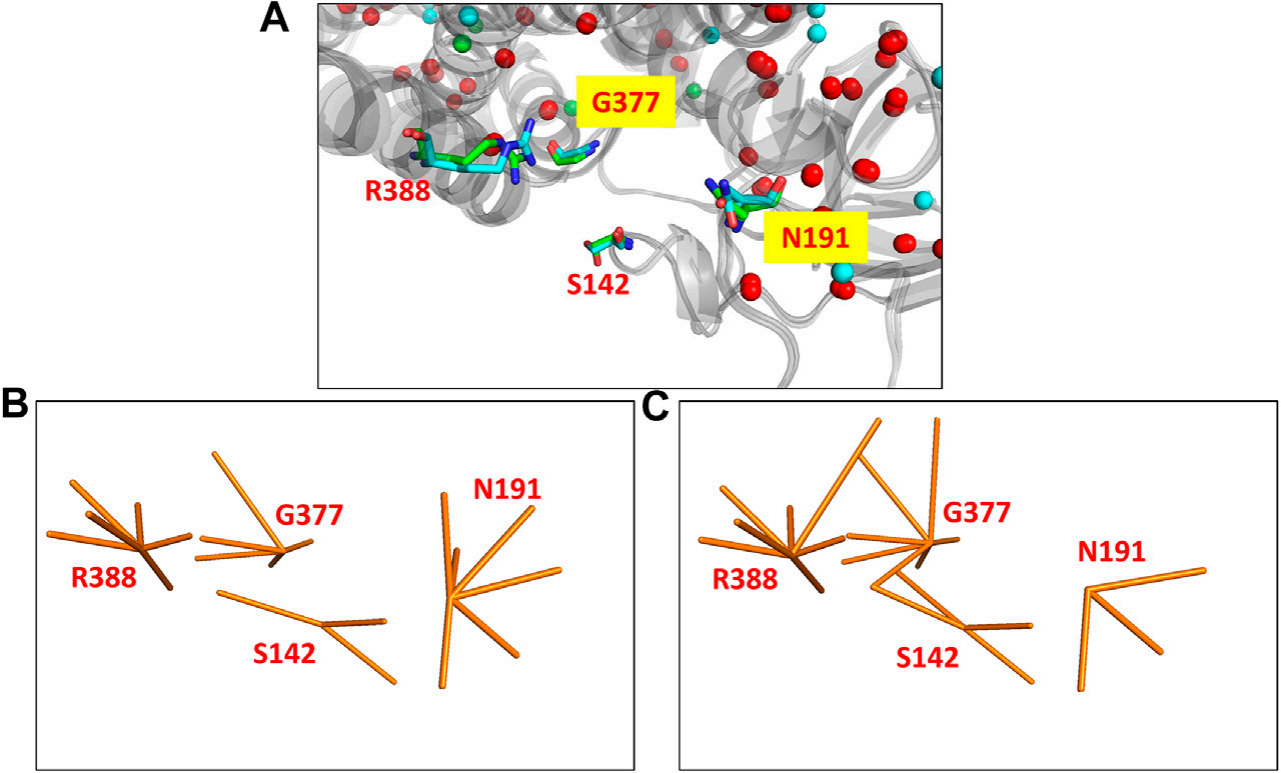
The functional site in the crystal structures of the wildtype (PDB ID: 1EGE) and mutant (PDB ID: 4P13) of the MCAD protein. **(A)** The functional site of the protein consists of four residues (S142, N191, G377, and R388) that are shown (using stick representation) in the superposed structures. The edges corresponding to these residues in the networks are shown in **(B)** the wildtype PSN and **(C)** the mutant PSN (using orange line representation). While N191 looses three edges, S142, G377, and R388 gain 1, 2, and 1 edges respectively.

The MCAD mitochondrial protein is known to catalyse the first step of fatty acid beta oxidation in humans. The functional protein is a homo-tetrameric complex with subunits bound to FAD molecules (Lee et al., 1996). The coding gene undergoes a single nucleotide polymorphism (A985G) that results in the protein mutant (K304E) which leads to the disease state (Gregersen et al., 1993). The protein undergoes a significant variation in the all-atom network (NDS 0.248), however the Cα RMSD is quite low (0.46Å). 67 edges and five hubs are lost in the wildtype PSN whereas 83 unique edges and 17 hubs are gained in the mutant (Supplementary Figure S8). It is observed that mutational site is far away from the site of function (S142, N191, G377, and R388). The site of function in the protein is shown in Figure 2A, the corresponding nodes and their edges in the wildtype PSN and mutant PSN are shown in Figures 2B,C respectively. Due to the rearrangement of edges at the nodes corresponding to functional site residues as shown in Figure 2, there is change in the local network at the functional site. It is reported that the mutation (K304E) leads to a deficiency of the protein that can result in death at infancy.

#### Porphobilinogen Deaminase Undergoes Disease Mutation That Leads to Loss of Essential Edges and has Reduced Thermostability

Porphobilinogen deaminase is a transferase that catalyses the synthesis of hydroxymethylbilane which is a precursor for heme and porphyrin biosynthesis. The disease mutant has defects of heme biosynthesis, which is mainly due to the enhanced excretion of porphyrins and porphyrin precursors. It is reported that the hydrogen bonding network in the ordered regions of the protein allows for the protein to display higher thermostability (Bustad et al., 2013). It has also been reported that the mutant crystal structure is less thermo stable and has lost its function and hence may be the leading cause for Acute intermittent porphyria (Gill et al., 2009). Although a significant number of edges and hubs are found to be preserved in the PSN, it is observed that 68 edges and 11 hubs that are unique to the wildtype is lost and 46 edges and three hubs unique to the mutant is gained (Supplementary Figure S9). Since there is loss of edges around the ordered secondary structures in the wildtype the important network necessary for thermostability is lost.

#### The Network Around the Functional Site in the Disease Mutant of Glutamine--tRNA Ligase Is Altered

The glutamine tRNA ligase is essential for the biosynthesis of glutamine in humans. The function of this protein is crucial for brain development in infants (Zhang et al., 2014; Ognjenović et al., 2016). The wildtype and mutant structures of the protein are well superposable (RMSD 0.68 Å) although their PSNs are quite dissimilar (NDS 0.24). The mutant node is far from the functional site where minimal variation of edges is observed. However, the significant loss of 176 edges and 32 hubs which are majorly found around the functional site in the wildtype PSN (Supplementary Figure S10) can be the cause for reduced aminoacylation activity reported in the mutant to cause microcephaly, progressive, with seizures and cerebral/cerebellar atrophy.

### Cases with Backbone Structure Variation

#### The Mutant Structure of the Major Prion Protein Undergoes a Conformational Switch

**FIGURE 3:**
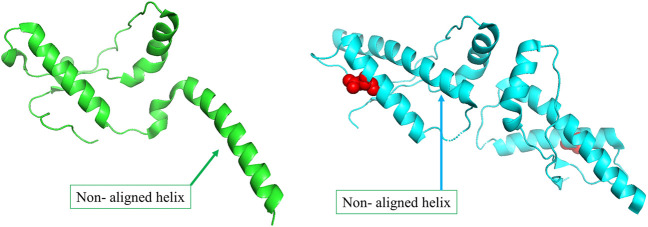
**(A)** The wildtype conformer (PDB ID: 1I4M) is crystallised as a monomer in the asymmetric unit, although it exists as a dimer functionally. **(B)**The structure of the disease-causing mutant (PDB ID: 3HEQ) shows conformational change in the non-aligned helix. The mutant residue is shown in red spheres.

The primary physiological function of the major prion protein is unclear. However, the functional state of the protein (Figure 3A) forms a well interacting dimer that is known to be involved in several different functions (Knaus et al., 2001). In the disease mutant state (Figure 3B), a conformational transition is observed in the C-terminal helix (Non-aligned helix shown in Figure 3) that forms a dimer with fewer interaction between the dimeric chains (Lee et al., 2010). The conformational change alters the topology at several other regions of the protein resulting in a high structural difference (RMSD 2.11 Å). However, the network in the topologically equivalent regions of the protein is preserved (NDS 0.153). There is only one hub in the wildtype that is not altered in the mutant and very few edges are rearranged, 19 edges and 23 edges unique to the wildtype and mutant respectively (Supplementary Figure S11). The new mutant conformation is found to be associated with Creutzfeldt-Jakob disease where cases are reported of degeneration of neurons and amyloid plaque formation due to protein aggregation.

#### Calmodulin-1 Mutant Acquires a Closed Conformation With Minimal Change in Network

Calmodulin is a membrane binding calcium transporter protein that transports metal ions across ion channels. A calcium ion binding sequence motif that occurs in pairs is conserved in the structures of this family of proteins (Tsang et al., 2006; Sarhan et al., 2012). There are two pairs of these binding site regions which are far apart in the open conformation of the wildtype structure. In the current case, when one of the calcium binding sites undergoes mutation (N98S), the functional state of the protein is lost (Wang et al., 2020). The mutant structure has a closed conformation which is reported not to bind to the metal ion at one of the calcium binding sites with the mutation. 21 edges in the wildtype and 18 edges in the mutant are lost and gained respectively. Seven hubs are retained and a single hub in the wildtype is lost in the mutant (Supplementary Figure S12). The overall network difference (NDS 0.121) is found to be minimal. However, due to the mutational site region that is found not to align well with the residues in the wildtype results in a large structural difference (RMSD 1.82 Å).

#### Structural Divergence in Wilms Tumour Protein

The Wilms tumour protein is a transcriptional factor consisting of a DNA binding domain which has four zinc finger repeats that determine sequence specific binding to DNA (Hamilton et al., 1995). While two of the zinc fingers bind to the DNA others are essential for recognising the cognate nucleotide base. One of these zinc fingers that is responsible for recognising the cognate nucleotide base undergoes a mutation (M342R) that enhances the affinity for a different nucleotide base leading to errors in transcription (Wang et al., 2018). The conformation of the wildtype does not superpose well with the mutant (RMSD 1.69 Å). In the PSN, 12 edges are lost in the wildtype and eight edges are gained in the mutant. One new hub is gained in the mutant along with the 1 hub that is retained between the wildtype and mutant PSN (Supplementary Figure S13). Hence, the network in the several regions of the protein is still preserved depicting low network dissimilarity (NDS 0.144).

### Allosteric Effect due to Disease Causing Mutation

**FIGURE 4:**
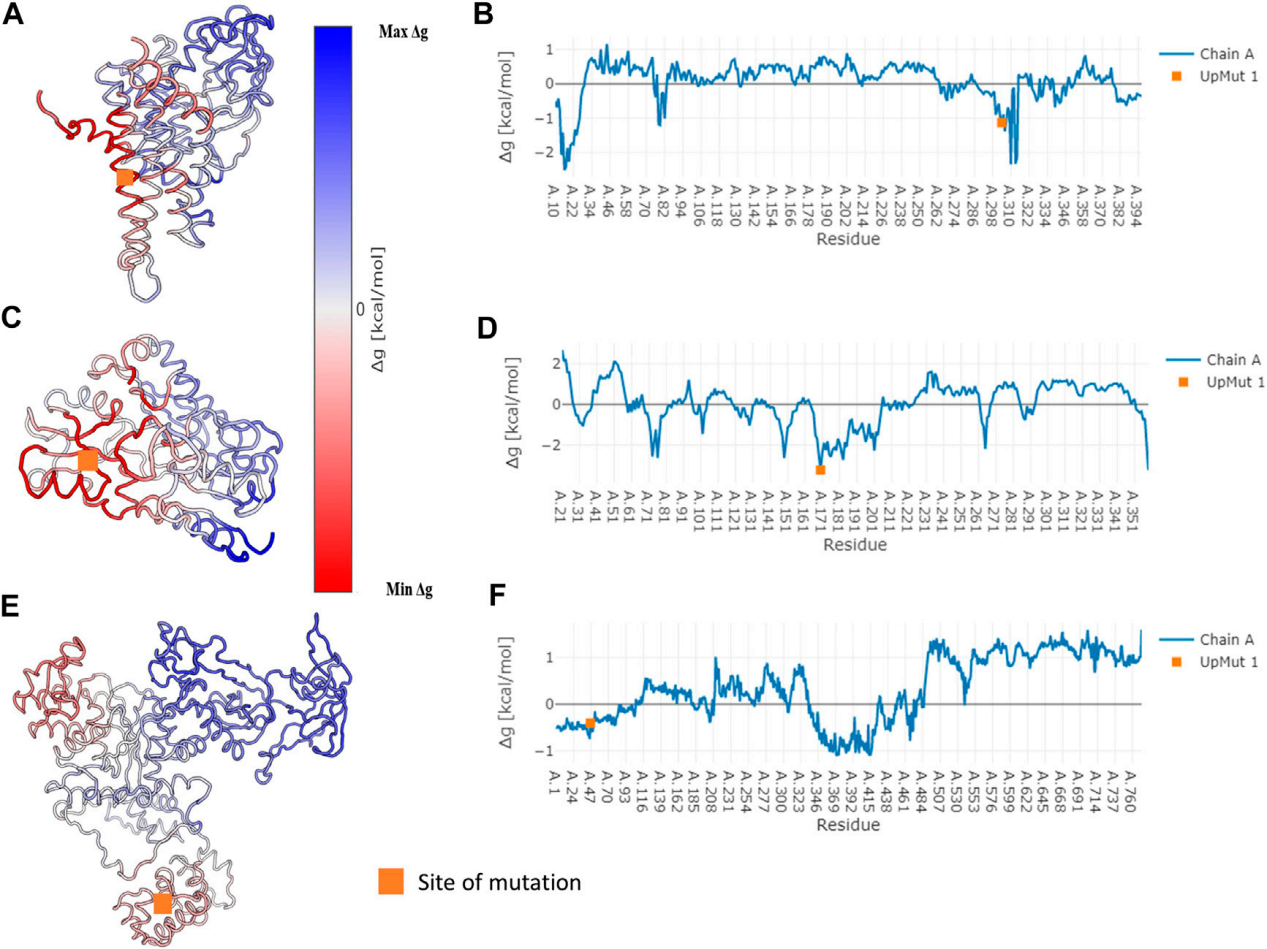
Free energy values obtained for three specific proteins that undergo disease-causing mutation. Specific cases where we observe significant network variability have been subject to the analysis of allosteric effects due to mutation. The AlloSigMA server employs the SBSMMA (Guarnera and Berezovsky, 2016) method to generate the response free energies when perturbations (UP mutation) are introduced at known sites of disease-causing mutations. Cartoon of the wildtype coloured according to their free energy values obtained for the cases of **(A)** Medium-chain specific acyl-CoA dehydrogenase, **(C)** Porphobilinogen deaminase and **(E)** Glutamine--tRNA ligase are shown on the left. Their free energy profiles are illustrated graphically with residue index on the *x*-axis and Δg value on the *y*-axis in **(B)**, **(D)** and **(F)** shown on the right in the same order. The orange square points to the site of mutation.

In specific cases where we observe network variation that is far from the site of mutation, we describe the possibility of observing an allosteric signal that repacks the residues resulting in the alteration of PSN. In order to corroborate the exhibition of allostery in these proteins AlloSIgMA (Tan et al., 2020) is employed to quantify the energetics compounding the allosteric effects of a mutation. Crystal structures of the wildtypes of three proteins in our dataset that undergo significant network change upon mutation were studied using AlloSIgMA and UP mutations (A perturbation that simulates the effect of mutation to a bulkier residue) at known disease-causing mutation sites are implemented. The output generated is illustrated in Figure 4 and discussed in the following section.

## Discussion

The protein structure network is an efficient tool in analysing allostery in the protein structure (Süel et al., 2003; Di Paola and Giuliani, 2015). In our study, we have analysed the variation of PSN brought about by disease causing mutations to the native functional protein. We have observed the variability in edges and hubs that are important parameters that make the protein structural network. We have identified edges and hubs that are unique to the wildtype structure that are lost in the mutant where new edges and hubs unique to the mutant structure are gained. The use of such information can be discussed with the help of an example.

**FIGURE 5:**
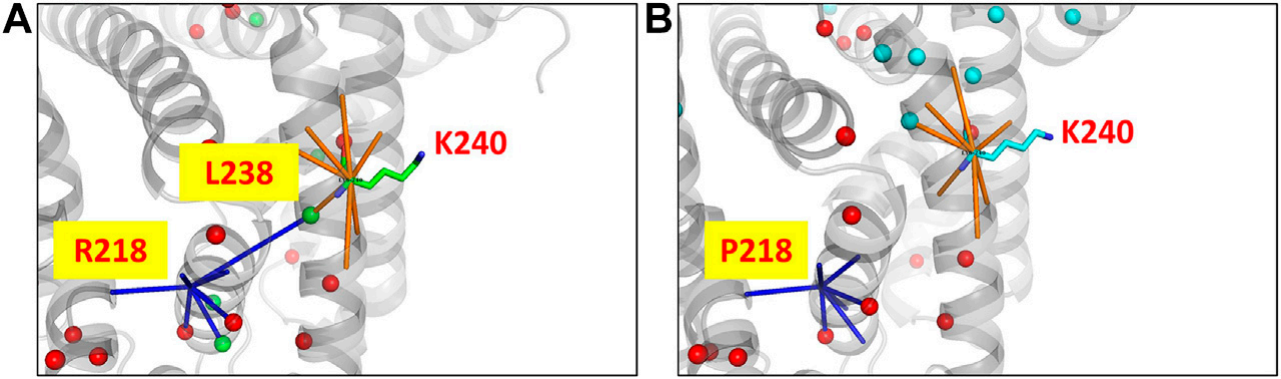
The PSN of human serum albumin protein at the site of mutation and function in the wildtype (PDB ID: 1N5U) and mutant (PDB ID: 1HK3) is shown. The node corresponding to the mutation site makes an edge with a hub node L238 (green sphere) in **(A)** the wildtype PSN which is lost in the case of **(B)** the mutant PSN. It is observed that hubs near to the binding site (K240) are lost, which is indicative of the increase in proximity between the nodes. It has been reported that the mutant structure is able to better accommodate a substrate with greater binding affinity which leads to the FDH disease condintion. Hubs unique to the wildtype and mutant are show in green and cyan sphere representation respectively, those hubs that are retained are shown in red.

The human serum albumin which is found abundantly in blood plasma is known to transport several different molecules including thyroxine (Robbins et al., 1978). In the dataset of disease cases, it is found that the mutant structure of albumin protein undergoes the largest variation in the number of edges and hubs. 294 edges and 25 hubs are lost in the wildtype and 305 edges and 20 hubs are gained in the mutant (Supplementary Figure S14). At the site of mutation (R218P) an edge with the residue L238 that is also a hub is found to be lost in the mutant (Figure 5). The loss of the edge is indicative of decrease in proximity between the residues suggesting that the thyroxine molecule that binds to K240, hormone binding site (Jacobsen, 1978), can be better accommodated in the mutant. It is reported that the mutation enhances the binding affinity of the protein to thyroxine that causes the elevated serum thyroxine levels associated with familial dysalbuminemic hyperthyroxinemia (FDH) (Petitpas et al., 2003).

We have analysed the variability in the disease cases by comparing their network and structure using the network dissimilarity score and RMSD. A control dataset is employed where the wildtype is compared to all other wildtype structures of the protein that satisfy the criteria for the dataset. The variability in disease cases (mean RMSD 0.92 Å and mean NDS 0.175) is much greater than in the variability in case of only wildtype structures (mean RMSD 0.57 Å and mean NDS 0.12) which signifies that the mutant structure and network explore diverse conformations with different interconnectivity of residues. The variability observed in protein structural networks is not strongly correlated to the topological structure difference that is used in the traditional analysis of protein structures. It is found that in a few cases, the network variability is relatively higher than the amount of structural difference. The vice versa is also true, where the structural difference is quite large but their networks seem to be well preserved. Such cases have been specifically picked for a detailed analysis of their global and local changes. We have also attempted to provide the functional relevance of the observed variability.

In the disease cases where the site of mutation is not involved with function, allosteric changes brought about in the connectivity of the internal network of the protein seem to affect the function which leads to a disease state. Where the contribution of the mutation may be as minimal as no change in the local network at the site of mutation, a large network alteration can be observed far away from the site of perturbation due to the disturbance in the network of edges connecting each element in the PSN to the other as discussed in the example of glutamine tRNA ligase. A significant improvement in the number of edges and hubs attributing to an improved network stabilises the MCAD protein although the distant mutation site alters the network at the functional site and hence the protein loses its function. Contrarily, a reduction in the number of edges and hubs in the case of the porphobilinogen deaminase protein is attributed to reduced thermostability due to loss of essential edges in the network within the protein. Conformational transition from one state to the other brings structural changes and loss of function in the case of major prion protein. However, their networks are found to be preserved since the aligned regions have retained edges and hubs that are very small in number. Likewise, it is found that there may not be a significant network variation but the structure varies considerably adding to the change in interaction with other molecule due to the mutation that eventually contributes to the alteration of function as observed in the case of Wilms tumour protein.

So as to substantiate the exhibition of allostery due to the mutations, theoretical free energy is computed using the AlloSigMA. The predicted free energy obtained for the specific cases of network variability when an UP mutation (mimicking substitution with a bulkier residue) is implemented at the site of disease-causing mutation are shown in Figure 4. A free energy value of zero suggests that the residue may not respond to the perturbation (mutation) whereas a non-zero value suggests that the residue may respond with more or less effect due to the perturbation. In the specific cases with large network variability, it is found that the disease-causing mutations stabilise (negative free energy) the residues around them and communicates the allosteric signal that destabilises (positive free energy) residues elsewhere within the structure. This suggests that the significant change in protein structural network that is observed due to the mutation at a site known to cause a disease is also due to the allosteric mechanism that arises from perturbation of the given site.

In Summary, our work highlights the perturbation of protein structural network as understood from the variability between a wildtype structure and the structure of a disease-causing mutant. Network features such as edges and hubs help to analyse the overall variation of networks while parameters such as degree of each node help to analyse their local network variability. The allostery due to a disease-causing mutation is noticeable from the loss and gain of network elements that result in variation of protein structural networks that is also corroborated using theoretical free energy calculations. We find cases where the network change is confined to the local site of mutation or far away from the site of mutation. We have also noted cases where repacking of sidechains occurs upon mutation and cases where the backbone conformation is altered with preserved sidechain network. From our work, the effect of mutation on the structural network of the wildtype may be used as a learning to extend to the next phase of the project to explore its predictive power of mutant structures and allosteric effects. The major challenge in the future is to translate the learning from the current work to predict the structure of the mutant which is a prerequisite to predict the effect of mutation on the stability and function. Availability of accurate structures of wildtype and reliably modelled mutant structures may be used in the context of thermodynamic cycle towards calculation of free energy difference between the wildtype and the mutant as for example used by Topham et al., (Topham, Srinivasan and Blundell, 1997). The protein structural network approach is an effective tool to understand the structural effects of disease-causing mutation, further we also suggest that the protein structural network approach is a convenient approach to understand the allostery caused by other kinds of structural perturbations.

## Data Availability

The original contributions presented in the study are included in the article/Supplementary Material, further inquiries can be directed to the corresponding author.
