# Supplementary material for: Influence of Disease-Causing Mutations on Protein Structural Networks
Source: Front Mol Biosci. 2021 Mar 10;7:620554. doi: 10.3389/fmolb.2020.620554 (PMC7987782; doi:10.3389/fmolb.2020.620554)
Supplement: Supplementary file 1 [file datasheet1.docx]

Supplementary Material

**1. Supplementary figures**


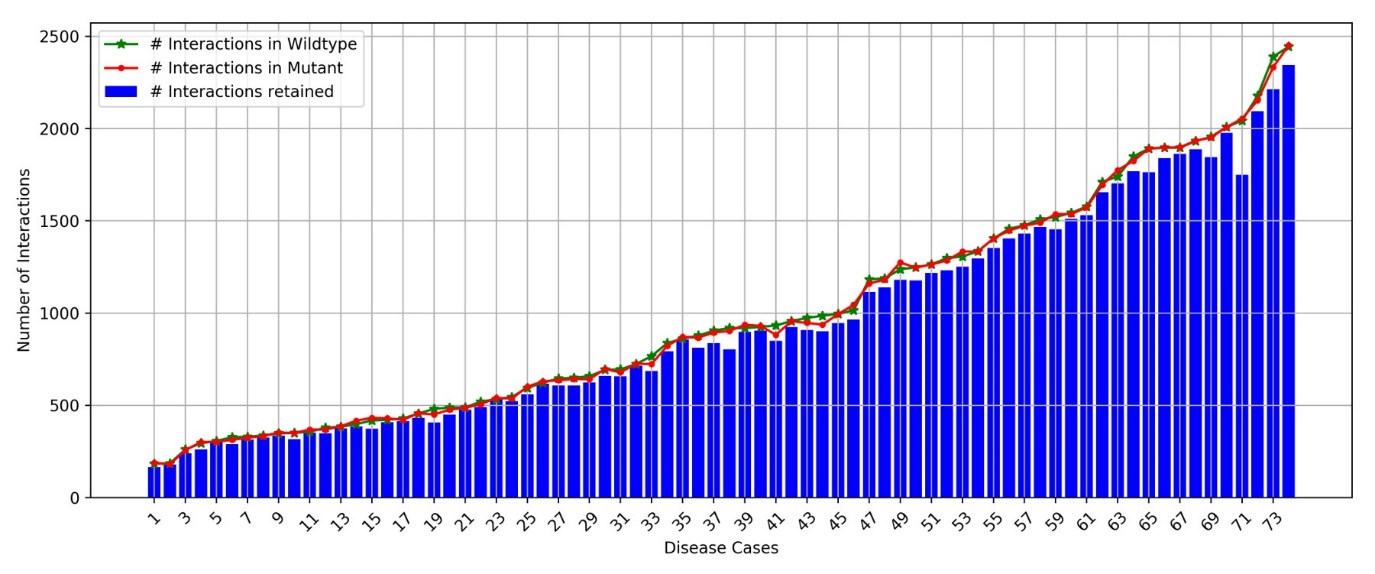


**Supplementary Figure 1:** Distribution of total number of edges in the PSN of wildtype and mutant shown using green and red plots respectively. Blue bars show the number of edges that are retained in the wildtype and mutant PSN. The distribution is arranged in ascending order of number of edges in wildtype of the disease cases.


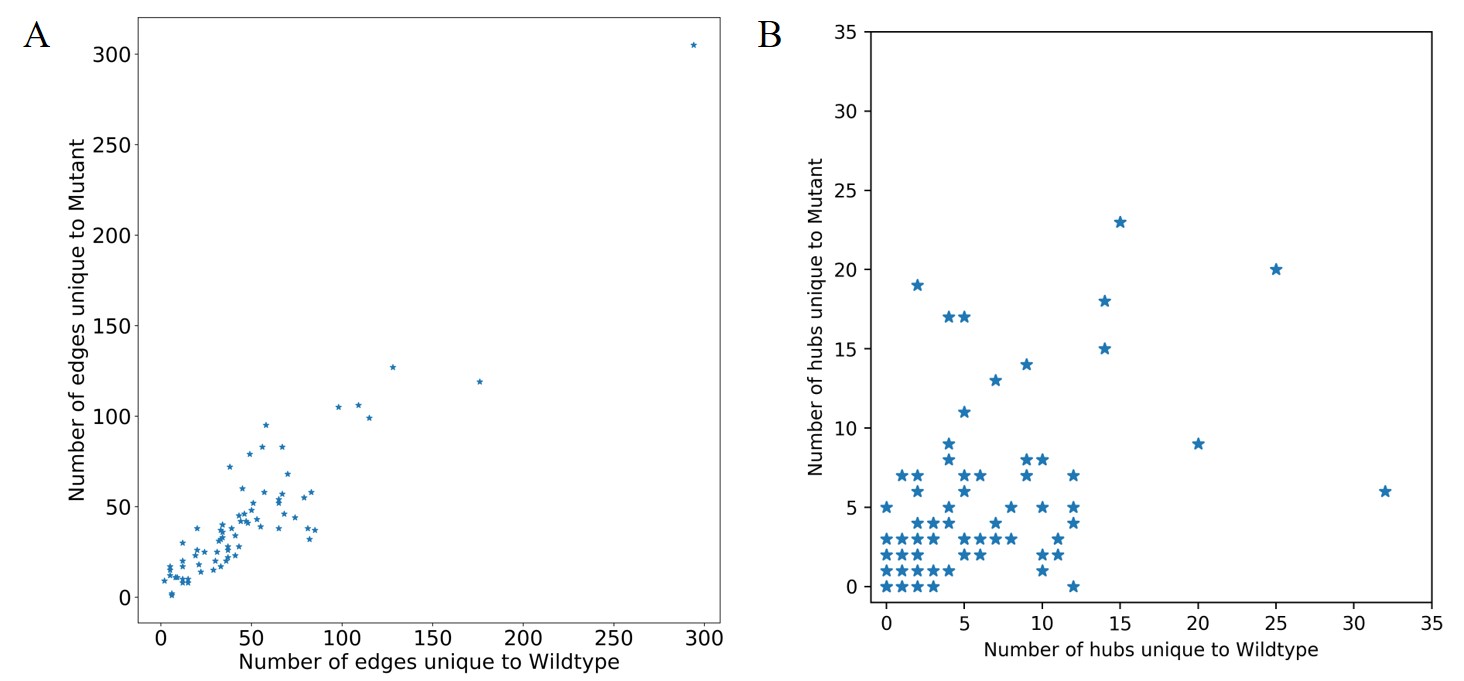


**Supplementary Figure 2:** Number of edges and hubs that are unique to the wildtype and mutant Protein Structural Networks (PSN). Those edges and hubs unique to the wildtype are considered to be lost in the mutant and those that are unique to the mutant are considered to be gained. **(A)** Scatter plot of the number of edges unique to the wildtype and mutant. **(B)** Scatter plot of the number of hubs unique to the wildtype and mutant.


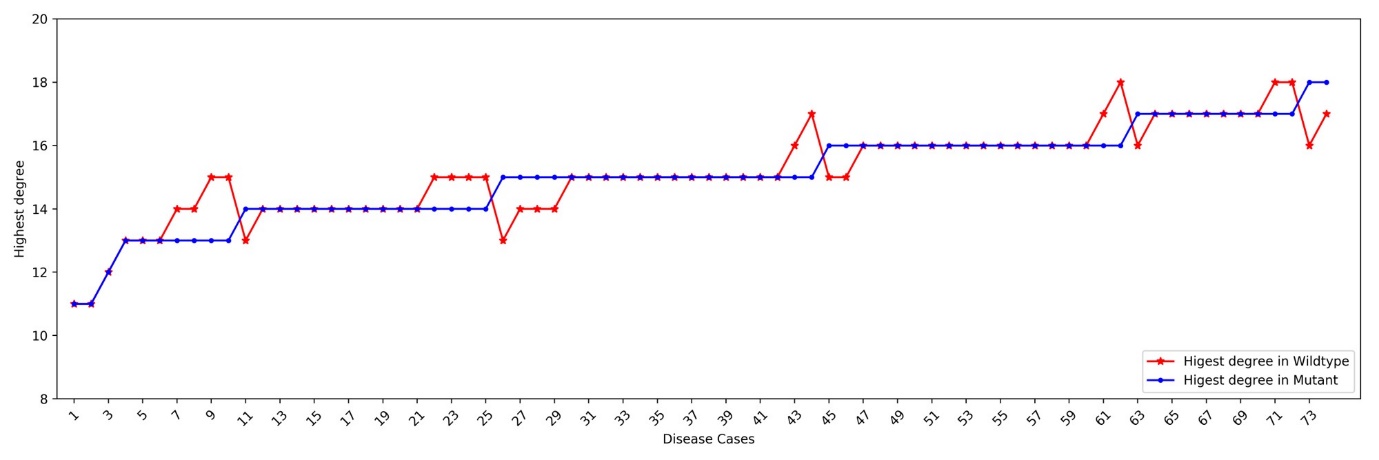


**Supplementary Figure 3:** The highest degree of a node that is found in the PSN of wildtype and corresponding mutant in each disease case. The range of the highest degree in the entire dataset is 11 to 18 hence we have used a degree cut-off of 11 to define hubs in our analysis.


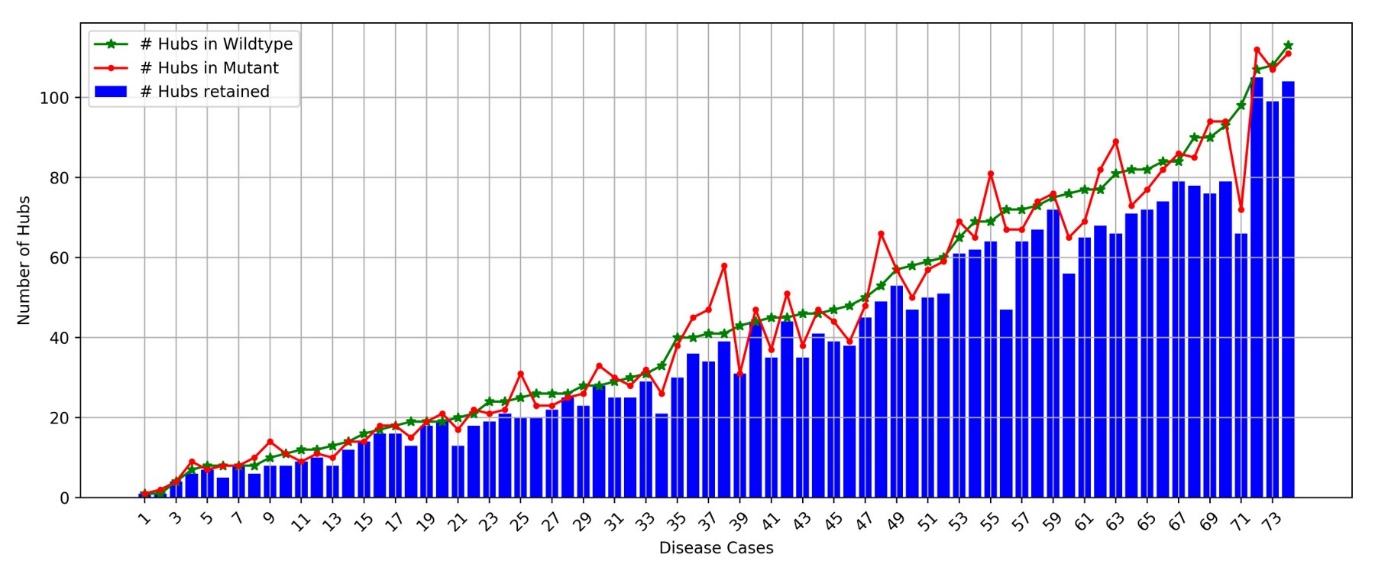


**Supplementary Figure 4:** Distribution of total number of hubs in the PSN of wildtype and mutant shown using green and red plots respectively. Blue bars show the number of hubs that are retained in the wildtype and mutant PSN. The distribution is arranged in ascending order of number of hubs in wildtype of the disease cases.


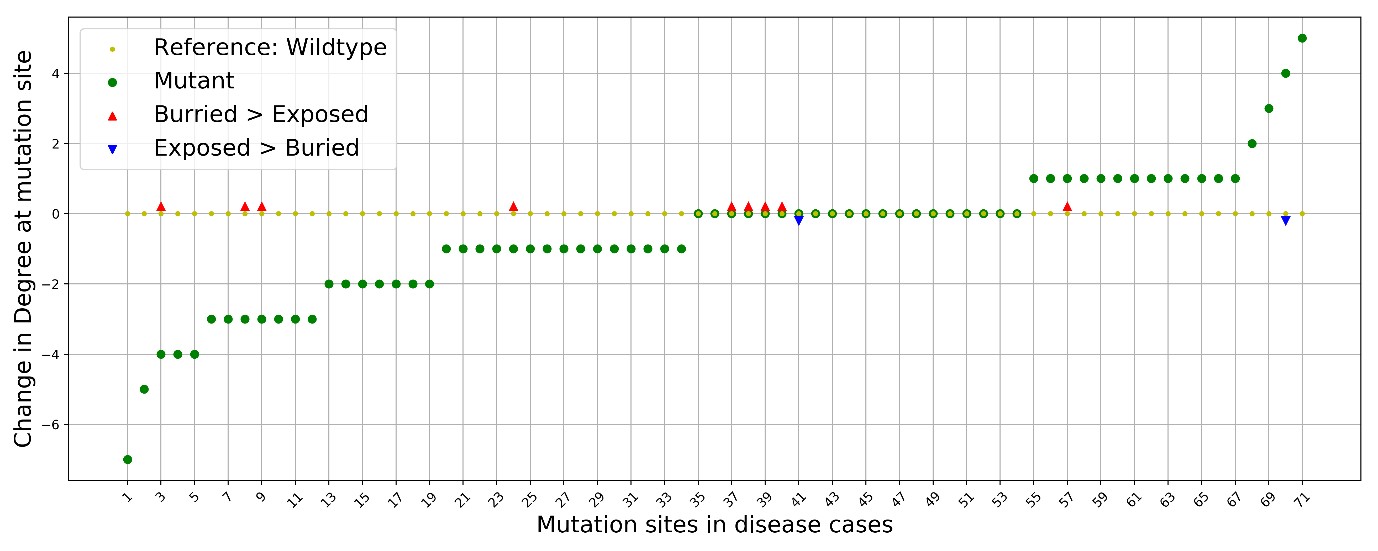


**Supplementary Figure 5:** The distribution of change in degree at the site of mutation in all the disease cases. The degree of the wildtype is used as a reference in calculating the change of the degree of the equivalent node in the mutant.


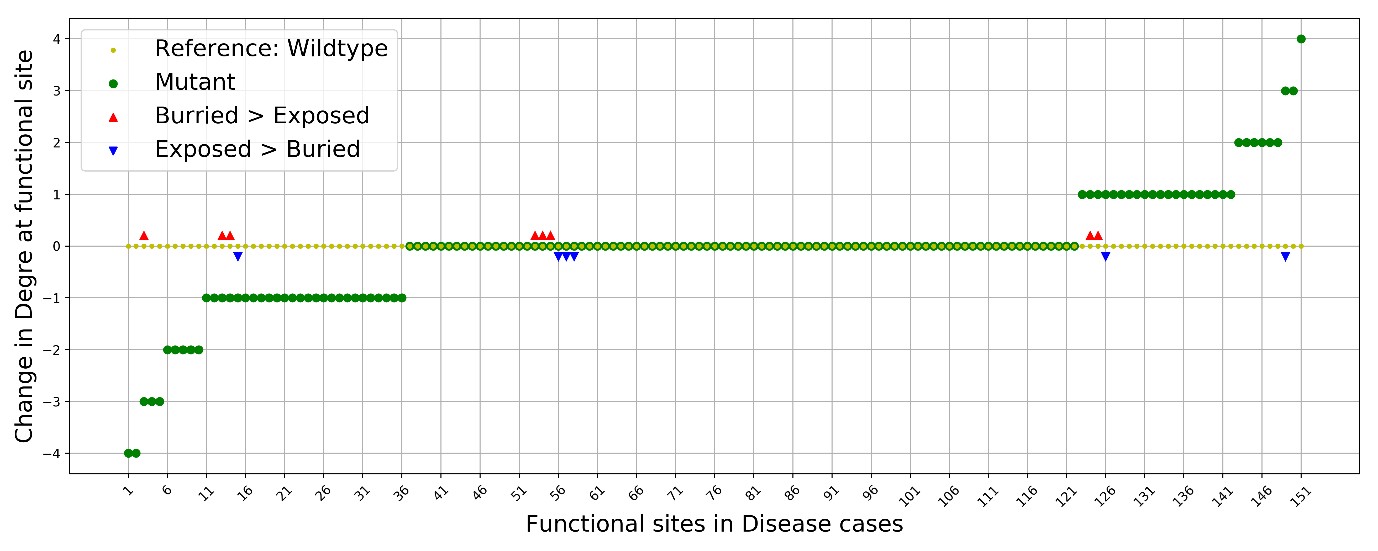


**Supplementary Figure 6:** The distribution of change in degree at the site of function in all the disease cases. The degree of the wildtype is used as a reference in calculating the change of the degree of the equivalent node in the mutant.


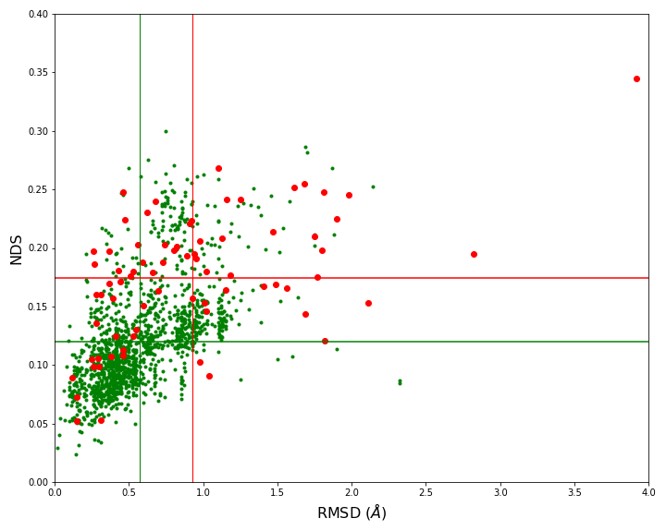


**Supplementary Figure 7:** The network and structural variability scatter obtained from the comparison of disease case wildtype and mutant crystal structures (shown using red dots) is plot along with the scatter of the control dataset where the wildtype structure is compared with all other wildtype structures of the protein that satisfy the criteria in the dataset (shown using green dots). The mean NDS and RMSD score of comparing the disease cases which is 0.175 and 0.92 Å respectively are shown using red lines and the mean RMSD and NDS score of comparing wildtype structures which is 0.12 and is 0.57 Å respectively are shown using green lines.


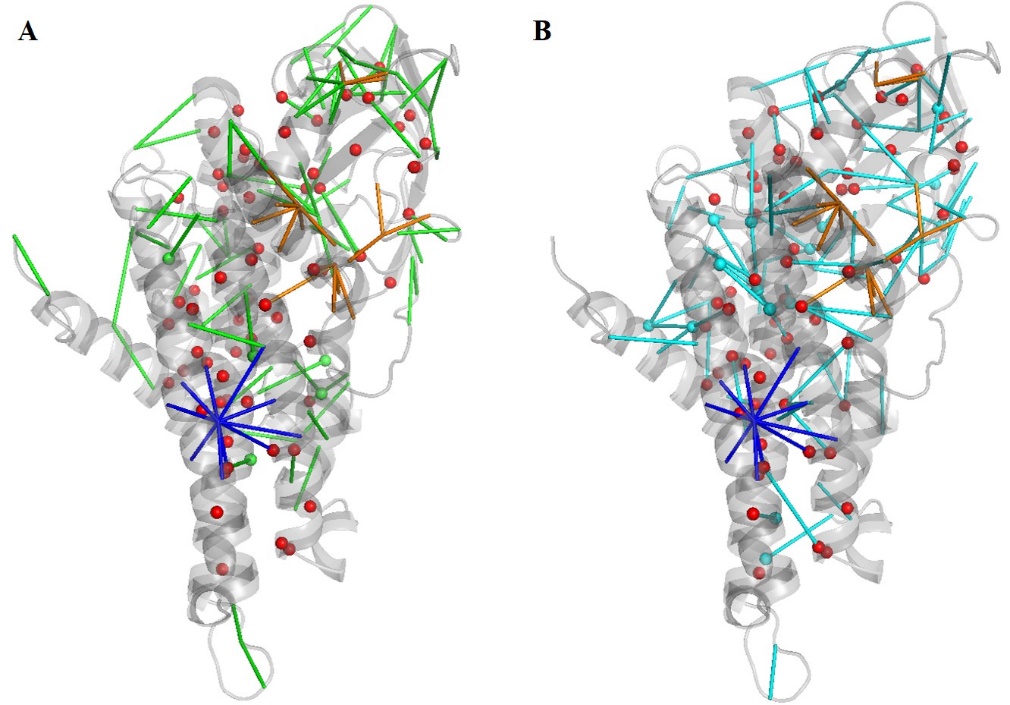


**Supplementary Figure 8:** Variation of network parameters in the **(A)** wildtype and **(B)** mutant structure of Medium-chain specific acyl-CoA dehydrogenase (MCAD). 67 edges and 5 hubs are lost in the wildtype whereas 83 edges and 17 hubs are gained in the mutant PSN. There is an increase in the number of edges and hubs of this disease case. The degree (10 edges) at the site of mutation (K304E) remains the same.


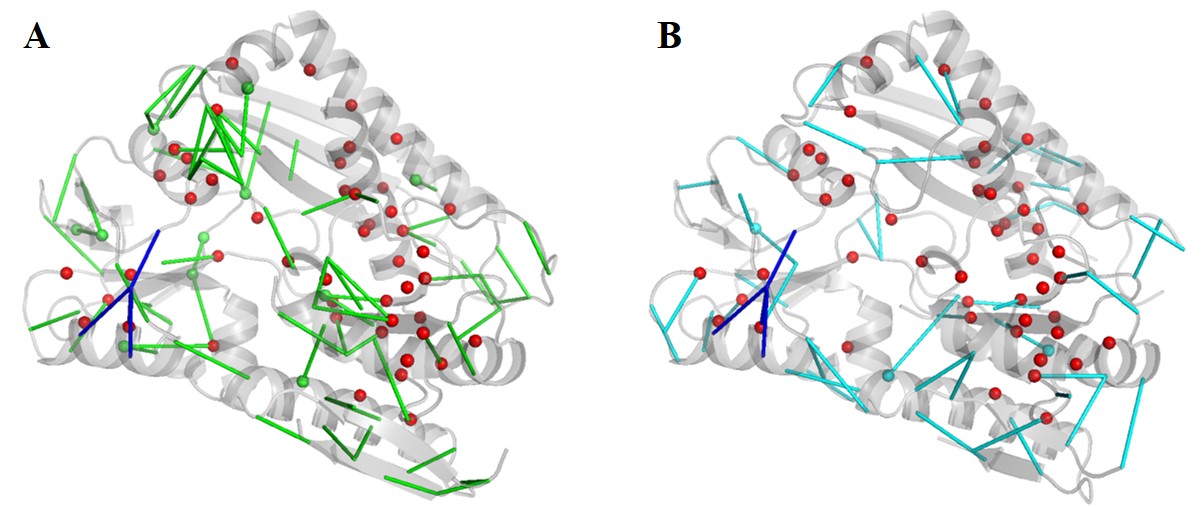


**Supplementary Figure 9:** Variation of network parameters in the **(A)** wildtype and **(B)** mutant structure of the Porphobilinogen deaminase. 68 edges and 11 hubs are lost in the wildtype whereas 46 edges and 3 hubs are gained in the mutant PSN. There is a drop in the number of edges and hubs of this disease case. The degree (4 edges) at the site of mutation (R170Q) does not change.


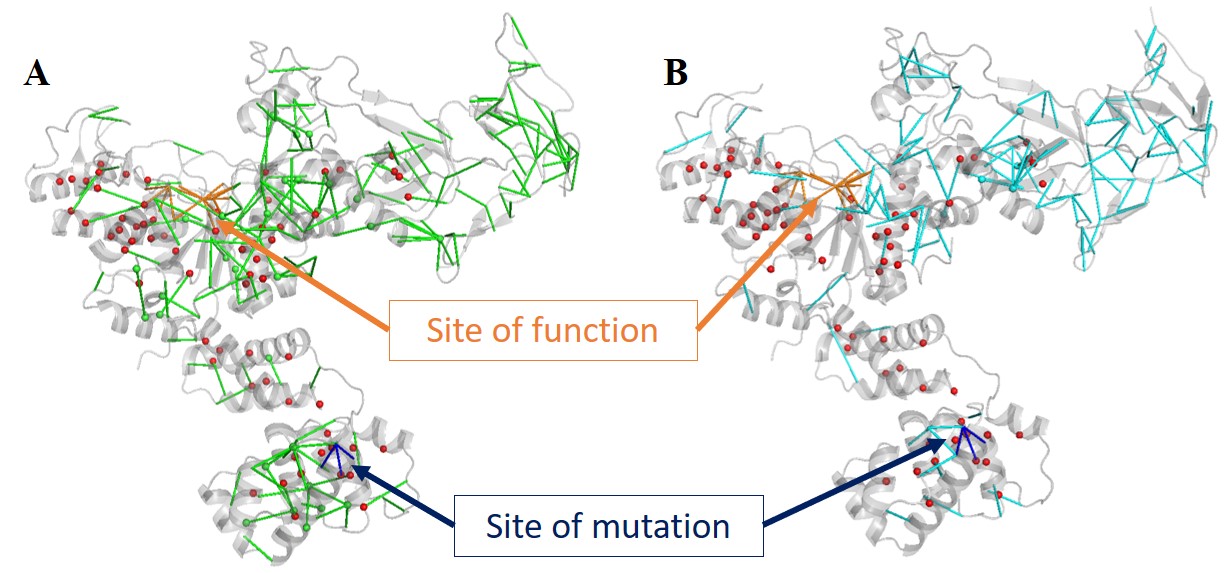


**Supplementary Figure 10:** The Glutamine tRNA ligase undergoes significant rearrangement of its network. The PSN of the **(A)** wildtype and **(B)** mutant are shown here. 176 edges and 32 hubs are lost in the wildtype whereas 119 edges and 6 hubs are gained in the mutant. The degree at the site of mutation (G45V) increases from 3 to 4.


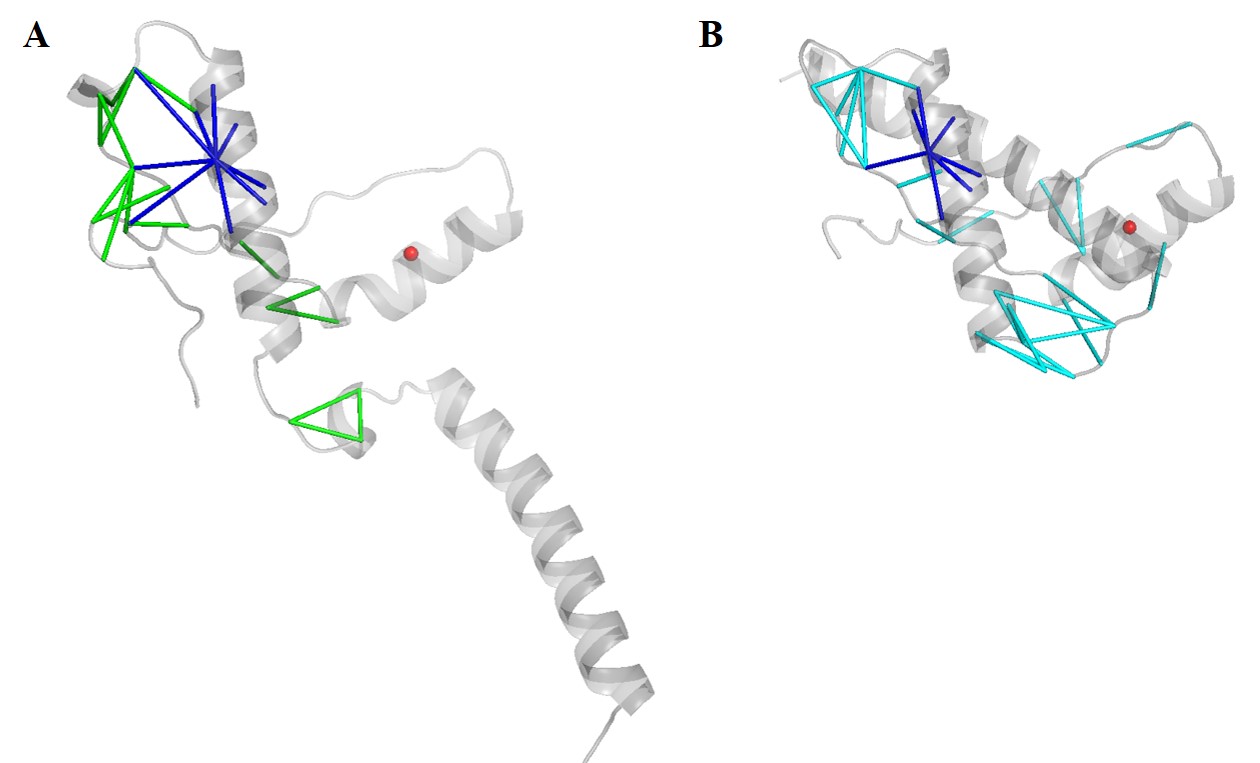


**Supplementary Figure 11:** Variation of network parameters in the **(A)** wildtype and **(B)** mutant of the major prion protein. A helix in the structure of the major prion protein undergoes a conformational change in the mutant that does not align with structure of the wildtype. Since the compared PSN includes only aligned regions of the protein the networks are found to be well preserved. However, 19 edges are found to be lost in the wildtype whereas 23 edges are gained in the mutant. There is only 1 hub in the PSN that is retained in both the structures.


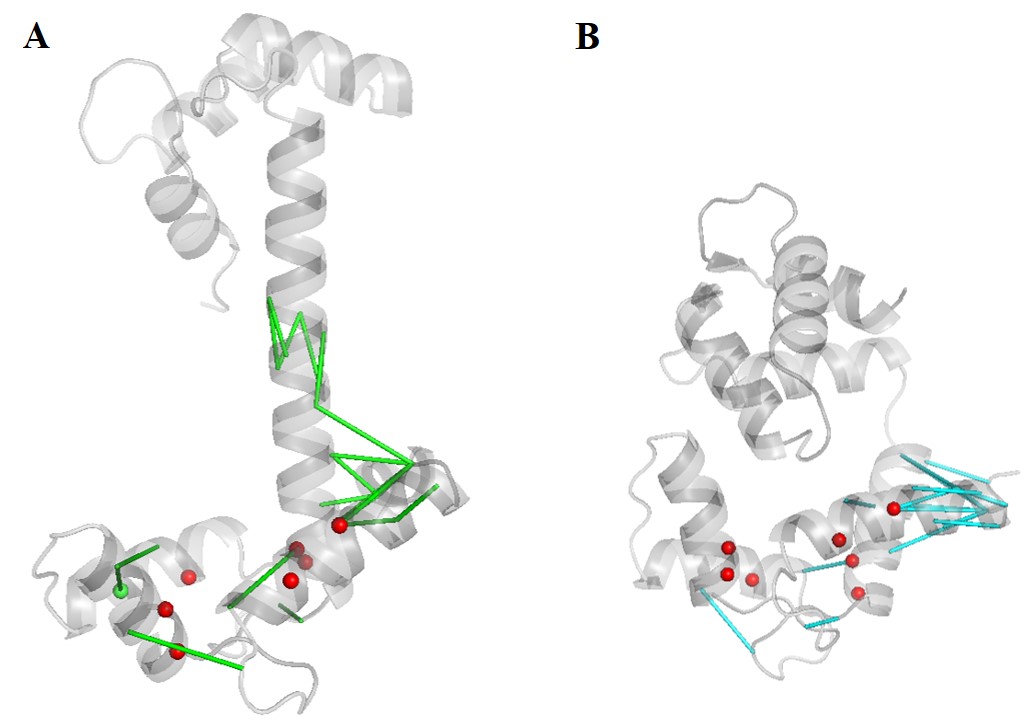


**Supplementary Figure 12:** Variation of network parameters in the **(A)** wildtype and **(B)** mutant of the Calmodulin protein. The wildtype structure of the calmodulin protein adopts an open conformational state whereas the mutant structure adopts a closed conformational state. The mutation site region of the mutant structure does not align with the wildtype structure. It is seen that the remaining region of the protein structure that aligns well, also has a retained network (NDS 0.121). 21 edges are lost in the wildtype whereas 18 edges are gained in the mutant. 1 hub is lost in the wildtype and no new hubs are gained in the mutant.


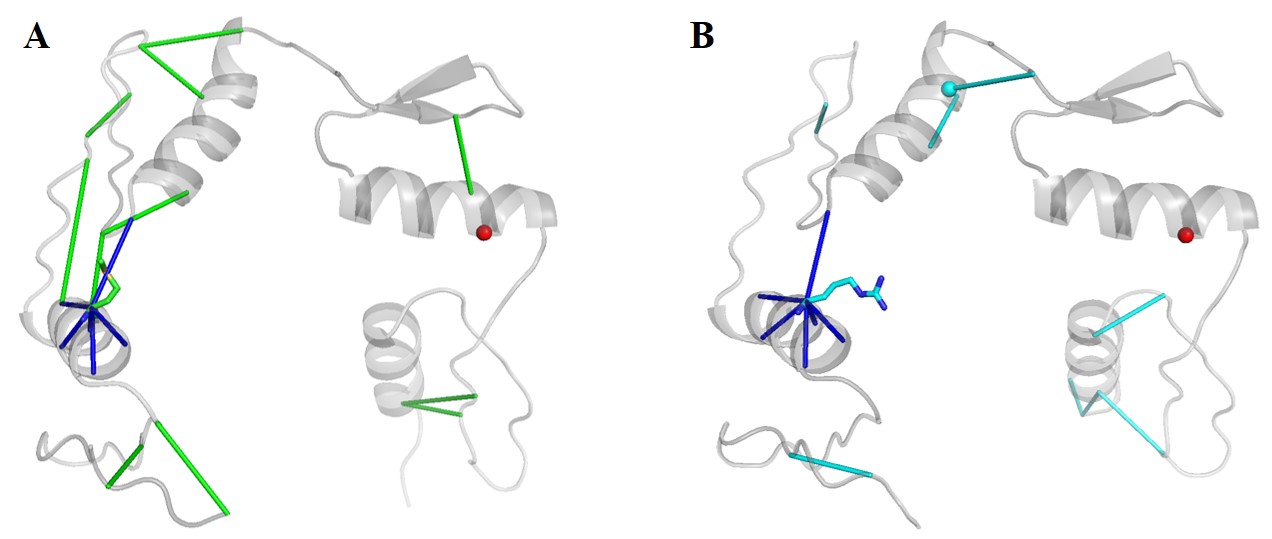


**Supplementary Figure 13:** Variation of network parameters in the **(A)** wildtype and **(B)** mutant of the Wilms tumour protein. 12 edges are lost in the wildtype whereas 8 edges are gained in the mutant. Only 1 hub is retained between the structures and 1 new hub is gained in the mutant.


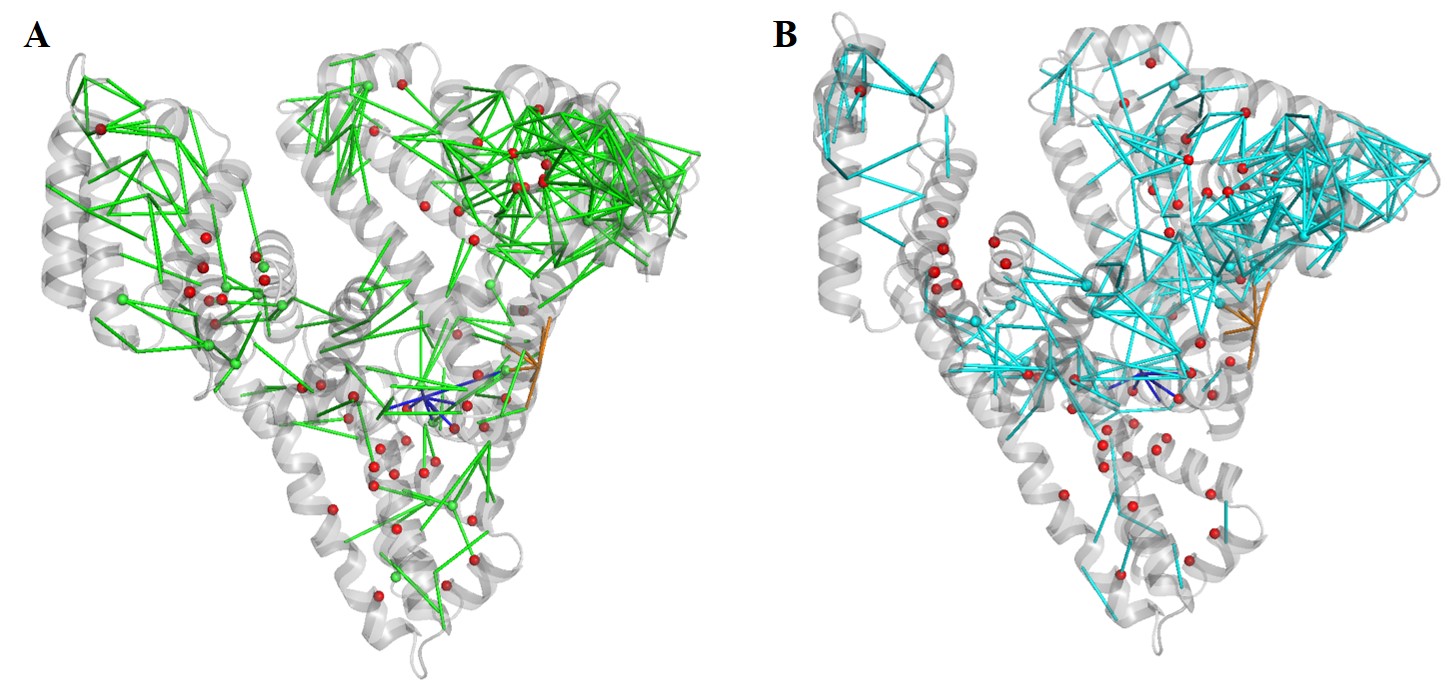


**Supplementary Figure 14:** Variation of network parameters in the **(A)** wildtype and **(B)** mutant of the human serum albumin protein. 294 edges and 25 hubs are lost in the wildtype whereas 305 edges and 20 hubs are gained in the mutant. The degree at the site of mutation (R218P) increases from 8 to 7.
